# Sphingomyelin Breakdown in T Cells: Role of Membrane Compartmentalization in T Cell Signaling and Interference by a Pathogen

**DOI:** 10.3389/fcell.2019.00152

**Published:** 2019-08-13

**Authors:** Elita Avota, Maria Nathalia de Lira, Sibylle Schneider-Schaulies

**Affiliations:** Institute for Virology and Immunobiology, Julius Maximilian University of Würzburg, Würzburg, Germany

**Keywords:** T cell, sphingomyelinase, activation, motility, measles virus

## Abstract

Sphingolipids are major components of cellular membranes, and at steady-state level, their metabolic fluxes are tightly controlled. On challenge by external signals, they undergo rapid turnover, which substantially affects the biophysical properties of membrane lipid and protein compartments and, consequently, signaling and morphodynamics. In T cells, external cues translate into formation of membrane microdomains where proximal signaling platforms essential for metabolic reprograming and cytoskeletal reorganization are organized. This review will focus on sphingomyelinases, which mediate sphingomyelin breakdown and ensuing ceramide release that have been implicated in T-cell viability and function. Acting at the sphingomyelin pool at the extrafacial or cytosolic leaflet of cellular membranes, acid and neutral sphingomyelinases organize ceramide-enriched membrane microdomains that regulate T-cell homeostatic activity and, upon stimulation, compartmentalize receptors, membrane proximal signaling complexes, and cytoskeletal dynamics as essential for initiating T-cell motility and interaction with endothelia and antigen-presenting cells. Prominent examples to be discussed in this review include death receptor family members, integrins, CD3, and CD28 and their associated signalosomes. Progress made with regard to experimental tools has greatly aided our understanding of the role of bioactive sphingolipids in T-cell biology at a molecular level and of targets explored by a model pathogen (measles virus) to specifically interfere with their physiological activity.

## Introduction

**FIGURE 1:**
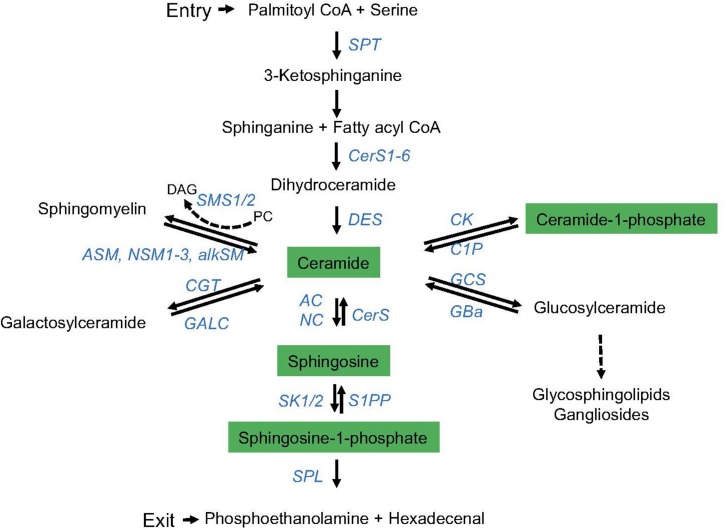
The sphingolipid metabolic pathway. *De novo* synthesis of endoplasmatic reticulum (ER) ceramide is initiated (entry) by serine palmitoyl transferase (SPT)-driven condensation of serine and palmitoyl-CoA, and further downstream activity of ceramide synthases (CerS1-6; giving rise to ceramides of different chain lengths) and desaturase (DES). Ceramide is reversibly converted into (1) sphingomyelin by sphingomyelin synthase 1 or 2 (SMS1/2) [reversed by acid (ASM), neutral (NSM, isoforms 1–3), or alkaline sphingomyelinases (alkSM)], (2) galactosylceramide [by galactosyltransferase (CGT) (reversed by galactosylceramidase (GALC))], (3) C1P by ceramide kinase (CK) [reversed by ceramide-1-phosphatase (CP)], (4) glucosylceramide by glucosylceramide synthase (GCS) [reversed by glucocerebrosidase (GBa)], or (5) sphingosine by acid or neutral ceramidase (AC, NC) (reversed by ceramide synthase, CerS). By phosphorylation, sphingosine kinases (SK1/2) generate S1P from sphingosine (reversed by S1P phosphatase, S1PP). S1P is irreversibly degraded into hexadecenal and ethanolamine by the activity of the S1P lyase (SPL) (exit of the sphingolipid metabolic pathway). Enzymes involved are marked in blue, and bioactive sphingolipids are highlighted in green.

Sphingolipids are abundant components of cellular membranes, and therefore, they effectively take part in cellular processes requiring membrane integrity and dynamics under rheostat and stimulated conditions (Airola and Hannun, 2013; Harayama and Riezman, 2018). Biosynthesis and metabolism of this particular class of membrane lipids are highly complex and subject to regulation by a variety of enzymes, and, except for initiation of synthesis and final hydrolysis of sphingosine-1-phosphate (S1P) into hexadecenal and phosphoethanolamine, reactions in this pathway are reversible. Thereby, many species of sphingolipids are generated that, in addition, vary in chain length, saturation, hydroxylation, and more or less complex head groups, altogether defining sphingolipid diversity (Hannun and Obeid, 2008; Gault et al., 2010; Feng et al., 2018) (Figure 1). Further expanding the complexity of the system, enzymes involved in sphingolipid metabolism may come in several isoforms differing in subcellular topology and cell type-specific expression levels (Clarke et al., 2006; Gault et al., 2010; Airola and Hannun, 2013). Therefore, biological outcomes of sphingolipid dynamics expectedly substantially differ.

Because of the high abundance of sphingolipid species, their composition within the plasma and organelle membranes and the dynamic alterations substantially impact on membrane biophysics. In context with other membrane lipids such as cholesterol, sphingolipids (also dependent on their acyl chain lengths) regulate membrane fluidity, which is important for membrane deformability during inward/outward vesiculation and also endo/exocytosis (Hannun and Obeid, 2008; Feng et al., 2018). In addition, membrane proteins and associated membrane proximal signaling components compartmentalize within membrane domains formed at steady-state conditions or in response to stimulation or metabolic signals. Classically, these were termed “lipid rafts” and/or—experimentally defined—detergent-resistant membrane (DRM) domains with sphingomyelin, glycosphingolipids, and cholesterol as major components (Simons and Gerl, 2010; Nakayama et al., 2018). Their composition can be dynamically altered upon signals, for instance, provided by sensing of intracellular stress or receptor ligation. Although as for most microdomains their exact lipid composition is still not clear (Harayama and Riezman, 2018), formation of ceramide-enriched membrane domains in the anticytosolic (extrafacial) membrane leaflet has been intensively studied. These are generated as a result of sphingomyelin breakdown by acid sphingomyelinase (ASM), which results in local release of ceramide (thereby eventually displacing cholesterol) that subsequently condense into ceramide-enriched domains that serve as platforms for signal relay and initiation, which often directly involves regulation of membrane proximal cytoskeletal dynamics (Gulbins et al., 2004; Bollinger et al., 2005; Gombos et al., 2006; Adada et al., 2013; Schneider-Schaulies and Schneider-Schaulies, 2013; Avota and Schneider-Schaulies, 2014). Ceramide-enriched membrane microdomains can be visualized using specific antibodies and, more recently, by redistribution of functionalized ceramide analogs also in T cells (Collenburg et al., 2016; Walter et al., 2016). On these, sizes and distribution of ceramide clusters under steady-state conditions and after application of exogenous bacterial sphingomyelinase were determined by *d*STORM technology (Burgert et al., 2017). Bioactive sphingolipids, i.e., mainly ceramide, ceramide-1-phosphate (C1P), sphingosine, and S1P act as signaling molecules themselves, and therefore, dynamic alterations of this pool directly regulate many cellular responses ranging from development and expansion to autophagy and apoptosis (Hannun and Obeid, 2008).

In common to that of other cell types, metabolism of membrane lipids also plays a key role in T cells as excellently reviewed (Wu et al., 2015, 2016; Howie et al., 2017). This review will focus on the role of sphingomyelin breakdown and subsequent ceramide release in various aspects of T-cell biology. Given the impact of this pathway on membrane and cytoskeletal dynamics and signaling in these highly motile cells, the activity of which relies on efficient propagation of external cues, this is particularly important. Rather than discussing the role of the bioactive sphingolipids themselves, this review aims at integrating the two most prominent, best-studied enzymes catalyzing sphingomyelin breakdown at the anticytosolic or cytosolic leaflet of the T-cell plasma membrane, the ASM and neutral (NSM2) sphingomyelinase.^1^

## Acid and Neutral Sphingomyelinases

**FIGURE 2:**
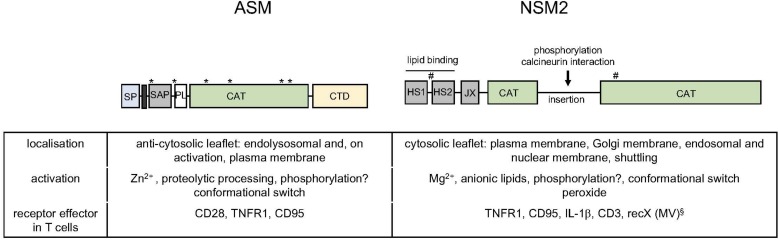
Acid and neutral sphingomyelinase 2: domains, subcellular distribution, and activation *in vitro* and in T cells. Schematic representation of functional domains within the ASM [signal peptide (SP), transmembrane domain (black bar), saposin domain (SAP), proline rich domain (PL), CAT and C-terminal domain (CTD) with glycosylation sites indicated by asterisks] and the NSM2 [hydrophobic segments 1 and 2 (HS1, HS2), juxtamembrane region (JX), and CAT, interspersed by an insertion that bears phosphorylation and protein interaction sites (including a calcineurin binding site); palmitoylation sites are indicated by hashtags] (Goni and Alonso, 2002; Airola and Hannun, 2013; Airola et al., 2017). The table summarized basic features of localization and activation of the enzymes and indicates receptors known to promote ASM or NSM2 activation in T cells. §: The identity of recX activating NSM2 in T cells by MV contact is as yet unknown (Gassert et al., 2009; Avota and Schneider-Schaulies, 2014; Mueller et al., 2014).

Isoforms of the ASM (encoded by the gene *SMPD1*) are secreted (sASM) or endolysosomal ASM, where its association to the anticytosolic membrane leaflet is required to protect the enzyme from degradation (Goni and Alonso, 2002). ASM activation is triggered by a variety of stimuli including cytokines, engagement of death receptors, or, as particularly relevant for this review, CD28. Receptor signaling-induced ASM activation can occur intracellularly after receptor signalosome fusion with endolysosomes (TNFR) or extracellularly after the enzyme is translocated from the internal storage compartment to the extrafacial leaflet of the plasma membrane (CD95) (Gulbins and Kolesnick, 2003; Henry et al., 2013) (Figure 2). There, ceramide release as a consequence of sphingomyelin hydrolysis promotes formation of ceramide-enriched membrane microdomains promoting formation and stabilization of receptor and signalosome complexes (Gulbins et al., 2004; Bollinger et al., 2005). At a biochemical level, ASM activation involves phosphorylation and proteolytic processing (Figure 2) (Henry et al., 2013). In addition to its role in cell activation, differentiation, and apoptosis, ASM has great importance for sphingolipid homeostasis as reflected by the development of severe, often fatal sphingolipidoses upon genetic ASM deficiency in mice and humans, where it is called Niemann–Pick disease (Horinouchi et al., 1995; Smith and Schuchman, 2008; Schuchman and Desnick, 2017). Though healthy at birth, ASM-deficient mice come down due to a progressive lysosomal sphingolipid storage as indicated by an increasing amount of foam cells in the bone marrow with age, extending to all visceral organs (Kuemmel et al., 1997). Activity of the ASM in the T-cell compartment has been clearly revealed where, and not surprisingly, ASM deficiency correlated with elevated levels of sphingomyelin (Boucher et al., 1995; Horinouchi et al., 1995; Herz et al., 2009; Tischner et al., 2011; Beyersdorf and Muller, 2015; Hollmann et al., 2016). ASM has been found to be particularly important in CD4^+^ regulatory T-cell (T*_*reg*_*) frequency and function in mice, indicating that the enzyme impacts on the composition of the T-cell compartment under homeostatic conditions (Hollmann et al., 2016; Zhou et al., 2016). An outer membrane-resident ASM-like enzyme, SMPDL3B, was identified in macrophages, where it reduced Toll-like receptor (TLR) responsiveness (Heinz et al., 2015). Though it may also be expressed in T cells, its function there is as yet unknown. Assuming it acts as a bona fide sphingomyelinase, it appears to do so at neutral rather than acidic pH (Heinz et al., 2015; Gorelik et al., 2016a,b) and could thereby be considered as neutral rather than ASM.

The best-studied member of neutral sphingomyelinases, NSM2 (*SMPD3-*encoded), is ubiquitously expressed and most abundant in the brain (from where it was initially purified) but also in lymphatic tissues (Liu et al., 1998). It is associated with cytosolic membrane leaflets of many compartments, including the plasma, Golgi, and endolysosomal and nuclear membranes (Stoffel et al., 2005; Albi et al., 2008; Cascianelli et al., 2008; Clarke et al., 2008; Trajkovic et al., 2008; Milhas et al., 2010b; Lucki and Sewer, 2012 Airola and Hannun, 2013; Stoffel et al., 2016) (Figure 2). Despite that its substrate is much less abundant at the cytosolic membrane leaflets, NSM2 is important in sphingolipid homeostasis, because in the absence of its activity, ceramide levels are up to 50% reduced (Marchesini et al., 2003; Stoffel et al., 2016). NSM2 is activated in response to stress signals and ligation of receptors such as TNFR1, CD95, CD40, and, as particularly relevant here, CD3 (Tonnetti et al., 1999; Clarke et al., 2006; Wu et al., 2010; Airola and Hannun, 2013; Shamseddine et al., 2015; Bortlein et al., 2018) (Figure 2). Shuttling between compartments and recruitment into receptor signalosomes are important in NSM2 activation (Clarke et al., 2008; Philipp et al., 2010). Its amino-terminal (NTD) hydrophobic domain marked by two conserved hydrophobic segments (HS1 and HS2) associates with, but does not span, the membrane; is separated from the cytosolic C-terminal catalytic domain (CAT) by a juxtamembrane domain, which interacts with the CAT domain upon phosphatidylserine (PS) binding to the NTD domain; and promotes a conformational switch needed for NSM2 activation (Figure 2) (Airola and Hannun, 2013; Airola et al., 2017). The extended CAT domain bears phosphorylation sites and a calcineurin interaction site that possibly regulate NSM2 activity and stability *in vivo* (Filosto et al., 2010, 2012; Shamseddine et al., 2015; Airola et al., 2017) (Figure 2).

Mice deficient for NSM2 activity due to the fragilis ossium (fro) mutation within *Smpd3* (*Smpd3*^*fro/fro*^) or genetic knockout (*Smpd3*-KO) reveal high embryonic lethality and, postnatally, severely impaired bone and tooth mineralization, skeletal deformation, and dwarfism, highlighting the importance of this enzyme as critical regulator of tissue development and homeostasis (Guenet et al., 1981; Aubin et al., 2005; Stoffel et al., 2005; Alebrahim et al., 2014). Limited availability of NSM2-deficient animals has as yet precluded studies on the impact of NSM2 deficiency on the lympho/monocytic compartment under homeostatic or infection conditions. Notably, mitochondrial ATP release has been shown to be regulated upon NSM2-driven ceramide generation, indicating that this enzyme takes part in metabolic homeostasis in astrocytes (Kong et al., 2018). If also applying to T cells, NSM2 activity could significantly impact on the kinetics and sustainment of T-cell activation and effector functions.

## Activation of ASM and NSM2 in T Cells

Though their impact on T-cell viability, expansion, and function has been extensively studied (see below), the molecular basis of ASM/NSM activation in these cells remains anecdotal. Non-overlapping motifs were identified in the cytosolic domains of TNFR and CD95, which independently regulate activation of ASM and NSM2. For the latter, this involves plasma membrane recruitment of nuclear EED/WAIT that bridges interacting NSM2 to the receptor-associated FAN (factor associated with NSM)/RACK1 complex also in Jurkat T cells (Philipp et al., 2010). Ligation of CD3 and CD28 is known to activate NSM2 and ASM, respectively (Boucher et al., 1995; Tonnetti et al., 1999; Mueller et al., 2014; Bortlein et al., 2018), but the underlying mechanisms are as yet unknown. In addition to its extensively studied role in NFAT activation in costimulated T cells, early recruitment of calcineurin to the T-cell receptor (TCR) complex was recently reported, where it took part in Lck activation, and it is tempting to speculate that NSM2, harboring a calcineurin interaction site, would be corecruited with calcineurin upon CD3 ligation (Filosto et al., 2010; Dutta et al., 2017). Cross-regulation of sphingomyelinases has also been observed. Thus, ASM elevation at both protein and activity levels was found enhanced in NSM2-deficient fibroblasts, but not T cells (Qin and Dawson, 2012; Mueller et al., 2014). Most interestingly, NSM2 activation was retained in costimulated T cells, while that of ASM was not (Mueller et al., 2014; Bortlein et al., 2018). Cross-regulation of sphingomyelinases was also evidenced in T cells after surface interaction of measles virus (MV) with an unknown receptor on the T cells, which promoted ASM activation downstream of NSM2 (Gassert et al., 2009; Avota et al., 2010).

## Impact of ASM/NSM on T-Cell Activity and Function

Dynamic reorganization of both membrane domains with regard to receptor and signalosome segregation and the cytoskeleton is subject to regulation by sphingomyelinase activity. The very same processes are of obvious importance in essential aspects of T-cell activation and function such as (1) motility and homing, (2) activation on antigen recognition in the context of organized immunological synapses (IS) and expansion, and (3) effector functions. The following sections will therefore review the progress made in the understanding of ASM/NSM2 contribution to these processes.

## Impact of ASM/NSM2 Activity on T-Cell Motility and Tissue Homing

T-cell migration and interaction with the endothelium during tissue homing are governed by cell and receptor polarization and signal relay provided by chemotactic gradients and adhesion molecules (Ley et al., 2007; Sadik and Luster, 2012). Filopodia formed at the leading edge of migrating T cells sense directional information, which is translated into dynamic assembly of actin filaments pushing the leading edge forward until membrane-tension-generated forces lead to their retraction (Bornschlogl, 2013; Leijnse et al., 2015; Leithner et al., 2016). When coupled to adhesion to the extracellular matrix, the retrograde actin flow generates cell movement, which is supported by stress fiber contraction and actin disassembly in the retracting uropod (Nordenfelt et al., 2016). On endothelial cells, T-cell rolling and polarization are followed by activation of β2-integrins, most prominently lymphocyte function-associated antigen 1 (LFA-1), that switch between high- and low-affinity states (Shulman et al., 2009; Nourshargh and Alon, 2014; Katsuno et al., 2015). T-cell transmigration through the endothelium relies on the interaction of activated LFA-1 with endothelial cell intercellular adhesion molecule 1 (ICAM-1), and integrin clusters arranged as focal adhesions, which are linked *via* talin, kindlin, and vinculin to the cytoskeleton (Moser et al., 2009; Hogg et al., 2011; Nourshargh and Alon, 2014).

Supporting important roles of sphingomyelinases activity in this process, ASM deficiency conferred protection against the development of experimental autoimmune encephalitis (EAE) in mice in which T-cell adhesion, blood–brain barrier disruption, and intracerebral infiltration of inflammatory cells were blocked (Becker et al., 2017). Similarly, NSM2 activity was found to contribute to rapid homing of CD4^+^ T cells *in vivo*. When exposed to an NSM inhibitor prior to adoptive transfer, these accumulated to lower levels in spleen and lymph nodes than solvent-treated control cells (Collenburg et al., 2017). Impaired navigational capacity of leukocytes in zebrafish larvae in the absence of FAN further supported the potential role of NSM in cellular motility (Boecke et al., 2012).

**FIGURE 3:**
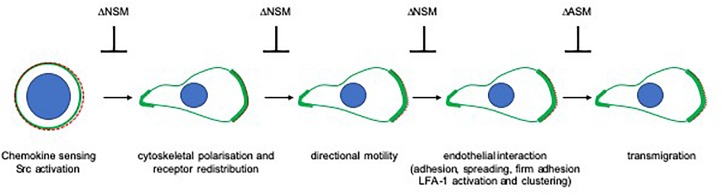
The role of sphingomyelinases in directional sensing and translation of chemotactic and endothelial signals in T cells. Perception of chemotactic signals through specific receptors (depicted as dashed red line) has not been found to be sensitive to ablation of NSM or ASM in T cells. Relay of these signals into morphological front-rear and actin (shown in green) polarization as well as receptor segregation to the leading edge is sensitive to NSM ablation as, consequently, are subsequent steps requiring directional sensing of external cues (directional migration, loose and firm adhesion, and spreading on epithelial cells as well as LFA-1 activation and clustering). In contrast, TEM of T cells relies on ASM rather than NSM activity. Notably, endothelial cell ASM is also required in this process.

Morphological polarization requires cytoskeletal dynamics. In the steady state, NSM2 may negatively control actin metabolism in T cells, because the average cell volume and frequencies of hairy-appearing, abundant protrusions are significantly elevated in NSM2-deficient Jurkat cells (Schoenauer et al., 2019). On stimulation, the role of NSM2 in T-cell morphology driven by actin reorganization may differ depending on receptor signaling: while NSM2 ablation in primary T cells enhanced spreading responses in response to CD3/CD28 engagement, morphological polarization of CD4^+^ T cells on fibronectin-coated surfaces and brain endothelial cells was effectively limited (Mueller et al., 2014; Collenburg et al., 2017) (Figure 3). While ASM deficiency did not detectably affect T-cell morphology, its hyperactivation in response to MV caused collapse of actin-based protrusions in ASM-sufficient cells (Gassert et al., 2009; Schoenauer et al., 2019). This could be rescued by ablation of the ASM, but also of the NSM2. In this particular activation system, NSM2 acts upstream of the ASM because both genetic and pharmacologic NSM inhibition prevent ceramide release at the cell surface (Gassert et al., 2009). Whether sphingomyelinase-driven actin cytoskeletal collapse observed in this system is specific for the receptor involved, signal strength, or the cell type is currently unknown as it has not been reported to occur upon ligation of classical activators including TNFR1, CD95, CD3, or CD28.

Morphological polarization associated with that of receptors is of obvious importance for spatial perception of chemoattractants. NSM2-deficient CD4 + T cells largely failed to polarize and to redistribute CXCR4 and thus, not surprisingly, were substantially impaired in directional migration and velocity in response to SDF-1α or in collagen matrices (mimicking extracellular matrix or tissue interaction) (Collenburg et al., 2017, 2018). Though not detectably affecting morphological polarization, pharmacological NSM2 inhibition also ablated directionality of human polymorphonuclear neutrophils (PMNs) in response to formyl methionine leucyl phenylalanine (FMLP), but not their overall motility (Sitrin et al., 2011). There, exogenous ceramide restored chemotactic responses indicating that loss of ceramide production due to loss of NSM2 activity causally related to inhibition of directionality (Sitrin et al., 2011). Exogenously added ceramides most likely incorporate into the outer leaflet, and therefore, compensation of ceramide loss at the inner leaflet would rely on flipping, which, at present, cannot be directly shown in living cells. Because SDF-1α and β1-integrins do not and FMLP is not known to activate NSM2, steady-state rather than stimulated activity of the enzyme is apparently required for morphological polarization of both CD4^+^ T cells and PMNs. There is, so far, no evidence that sphingomyelinases contribute to chemokine receptor or β1-integrin signaling directly, and therefore, impaired directional motility of NSM2-deficient T cells most likely reflects their inability to spatially perceive exogenous signals.

Failure to perceive exogenous signals may also be of crucial importance for explaining impaired adhesion of NSM2-deficient cells to interferon-γ (IFN-γ)/tumor necrosis factor-α (TNF-α)-activated endothelial cells both under static and shear stress conditions (Collenburg et al., 2017), which, as crawling and *trans*-endothelial migration (TEM), again depends on the relay of chemokine signals promoting polarization followed by integrin affinity maturation. In line with polarizing signals and associated actin reorganization being a prerequisite for firm adhesion to endothelial cells, NSM2-deficient CD4^+^ T cells seeded on brain endothelial cells failed to promote formation of activated LFA-1 clusters, which could not be stabilized by ICAM-1 clustered on endothelial cells. Possibly, sphingomyelinase activity may also directly impact on formation of activated LFA-1 clusters. Not shown to apply to T cells, LFA-1 clusters were found trapped in ceramide-enriched membrane domains on epithelial cells (Grassme et al., 2017), and exogenously provided ASM caused β1-integrin activation or modulated LFA-1-dependent adhesion to ICAM-1 (Carpinteiro et al., 2016; Eich et al., 2016).

In endothelial cells, ICAM-1 clustering and cytoskeletal anchoring as required for TEM was found to be dependent on ASM activity, which, in contrast to that of NSM2, was also required for transmigration in both endothelial and T cells (Lopes Pinheiro et al., 2016; Collenburg et al., 2017). Because NSM2 and ASM regulate cytoskeletal dynamics, their differential roles in T-cell polarization and transmigration most likely reflect differential cytoskeletal processes involved. Thus, pushing forces for scanning endothelial cells for suitable TEM spots, formation of focal adhesions and uropod contraction driven by microtubule depolymerization and increased RhoA/ROCK activity (Takesono et al., 2010) may be promoted by ASM activity (Chang et al., 2008; Stroka et al., 2013), while acquisition of a migratory, highly polarized lamellipodia/uropod-based phenotype in T cells rather relies on NSM2 activity (Figure 3).

## Sphingomyelinases in TCR Signaling, Costimulation, and Expansion

Immunological synapses formation requires attachment of scanning T cells followed by spreading to allow for functional organization of the antigen-presenting cell (APC)/T-cell interface. As detailed above, NSM2 supported T-cell attachment to endothelia, and it is unknown as yet whether it also takes part in mediating or stabilizing APC/T-cell conjugate formation. The latter has been found to be unstable when T cells were exposed to MV prior to dendritic cell (DC) conjugation (Shishkova et al., 2007), and though not experimentally addressed in this study, NSM2/ASM-mediated paralysis of the actin cytoskeleton might contribute to IS destabilization. Surface-bound chemokines are also important in capturing and priming T cells for synapse formation (Friedman et al., 2006), which, as described above, might be less efficient for NSM2-deficient cells. Conceivably, timing and magnitude of sphingomyelinase activation might define to what extent these enzymes take part in conjugate formation.

**FIGURE 4:**
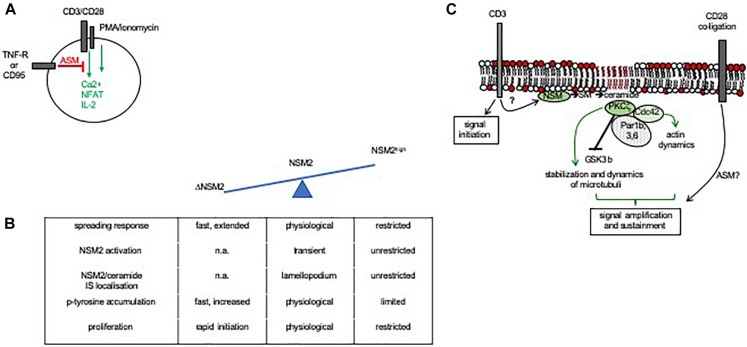
Role of sphingomyelinase activity in IS formation and T-cell activation. **(A)** ASM activation through death receptor signaling abrogated Ca^2+^ mobilization, NFAT activation, and IL-2 synthesis in costimulated or PMA/ionomycin-activated primary or Jurkat T cells (Lepple-Wienhues et al., 1999; Church et al., 2005). **(B)** In CD3/CD28 costimulated T cells, NSM2 activity is spatiotemporally tightly controlled under physiological conditions to promote actin cytoskeletal rearrangements required for IS formation (spreading response) and activity (as indicated by kinetics and magnitude of accumulating p-tyrosine protein species and proliferation). NSM2 hyperactivation in response to MV exposure causes loss of actin remodeling, enzyme and ceramide confinement to the lamellopodium, and impairment of IS activity, while NSM2 deficiency is associated with exaggerated cytoskeletal remodeling and early T-cell activation (Mueller et al., 2014). **(C)** NSM2 activation in response to CD3 ligation takes part in early TCR signaling. Though capable of initiating a first wave of TCR signaling, NSM2-deficient cells are unable to recruit a polarity complex (in T cells confirmed for PKCζ and Cdc42, not yet for Par1b, 3, and 6) and subsequently, the actin and microtubular system required for signal amplification and sustainment. NSM2 activity in TCR signaling is particularly relevant at low antigen doses, and in its absence, T-cell activation is highly dependent on signaling provided through costimulation. Whether or not ASM is also activated in costimulation is not entirely resolved as yet (Bai et al., 2015; Bortlein et al., 2018).

Much more is known about their role in the efficiency of T-cell activation. As for other cell types, ceramide release in response to death receptor ligation induces apoptosis in T cells (Andrieu-Abadie and Levade, 2002; Grassme et al., 2003; Detre et al., 2006; Edelmann et al., 2011), and especially for that of CD95, this contributes to activation-induced cell death and, thereby, homeostasis of the T-cell compartment. TNFR1 and CD95 ligation also inhibited mitogen-, αCD3/CD28-, or phorbol ester/ionomycin-stimulated Ca^2+^ influx, NFAT activation, and IL-2 synthesis in lymphoblasts and Jurkat cells *via* ASM induction, indicating that ceramide release catalyzed through its activity interferes with T-cell activation (Lepple-Wienhues et al., 1999; Church et al., 2005) (Figure 4A). In line with this hypothesis, accumulation of ceramides as measured in CD4^+^ T-cell IS and non-IS membrane fractions of aging mice correlated with decreased proliferative responses (Molano et al., 2012). In turn, NSM2-deficient cells proved to be hyperresponsive to αCD3/CD28 costimulation with regard to cytoskeletal reorganization, Ca^2+^ mobilization, and initiation of proliferation, and NSM2 activation at least partially accounted for the loss of T-cell proliferation induced through MV surface exposure (Mueller et al., 2014) (Figure 4B), altogether indicating that sphingomyelinase-mediated ceramide release might be inhibitory to T-cell activation, irrespective as to whether it is generated in the extrafacial or cytosolic leaflet of the plasma membrane. Notably, ceramide was found largely excluded from T-cell plasma membrane domains engaged in TCR signaling (Zech et al., 2009).

Strikingly, ligation of CD3 or CD28 by antibodies individually was found to cause activation of NSM2 or ASM, respectively. Interestingly, only NSM2 activation was retained upon αCD3/CD28 coligation by antibodies, and both NSM2 protein and ceramides accumulated in the lamellopodial area of the IS (though excluded from its center and the lamellum), indicating that sphingomyelin breakdown, if spatiotemporally controlled, may indeed take part in T-cell activation (Boucher et al., 1995; Tonnetti et al., 1999; Mueller et al., 2014; Bortlein et al., 2018) (Figure 4B).

In support of its importance in TCR signaling, NSM2-deficient cells proved to be highly dependent on costimulation and unable to mobilize Ca^2+^ at low (most likely physiologically more relevant) antigen doses (Bortlein et al., 2018). Acting at the cytosolic leaflet of the plasma membrane, NSM2 could potentially be involved in modulating the microenvironment of the TCR by possibly facilitating exposure of the CD3β chain, formation of TCR microclusters, displacement of cholesterol from the TCRβ chain, or Lck recruitment (Gagnon et al., 2012; Kapoor-Kaushik et al., 2016; Swamy et al., 2016; Pageon et al., 2016). However, neither formation of CD3 clusters nor activation of Lck was found to be affected in Jurkat cells deficient for NSM2, whose CD3-dependent activation occurred downstream of Lck, indicating that NSM2 might not be required for initiation of TCR signaling but rather its sustainment (Bortlein et al., 2018). This involves TCR microcluster trafficking, endocytosis, and directional exocytosis at the peripheral IS (Fooksman et al., 2010; Hashimoto-Tane et al., 2011). Moreover, production of TCR-containing vesicles and their release into the synaptic cleft have been found to enhance T-cell activation (Choudhuri et al., 2014). Though both NSM2 and ASM are known to regulate both cytoskeletal activity and endo/exocytosis (Draeger and Babiychuk, 2013; Schoenauer et al., 2019) (see also below), their involvement in these particular features of the mature IS has not been revealed as yet. In contrast, NSM-dependent formation and polarized release of exosomes from T cells toward the APC were described, revealing that this enzyme indeed contributes to communication at the level of the IS (Mittelbrunn et al., 2011).

Sustainment of TCR signaling relies on cytoskeletal reorganization and rapid polarization of organelles promoting the activity of the IS such as the microtubule organizing center (MTOC), Golgi, and mitochondria (Martin-Cofreces et al., 2008; Martin-Cofreces et al., 2014). In line with the key role of NSM2 in this process, NSM2-deficient cells, though capable of signal initiation, were unable to sustain Lck tyr398 phosphorylation and largely failed to polarize CD3 molecules and relocalize the MTOC in response to TCR ligation (Figure 4C). As reported previously in non-T cells (Krishnamurthy et al., 2007; Bieberich, 2011), NSM2 promoted membrane recruitment and activation of the atypical PKCζ, which proved to be crucial to support MTOC IS polarization. Exogenously applied ceramide efficiently rescued PKCζ membrane recruitment and MTOC polarization, indicating that formation of ceramide-enriched domains at the inner leaflet required for PKCζ activation was ablated in NSM2-deficient T cells. In line with previously reported microtubular stabilization due to either NSM2 or ASM activity as well as physical interaction of ceramide and tubuli, NSM2 deficiency resulted in microtubular destabilization also in T cells (He et al., 2012, 2014; Bortlein et al., 2018; Kong et al., 2018) (Figure 4C).

Though there is a clear role for NSM2 in TCR signaling, it is dispensable at high antigen dose and strong costimulation through CD28 (Mueller et al., 2014; Bortlein et al., 2018). The role of ASM in regulation of T-cell functions is multifaceted as it is reported to modulate TCR signaling *via* engagement of inhibitory (TNFR) and costimulatory (CD28) receptors. CD28 signaling pathways in costimulated T cells have been extensively refereed and will not be reiterated herein. We rather focus on the potential role of ASM activation seen upon engagement of CD28 for T-cell activation (Boucher et al., 1995; Collenburg et al., 2016; Bortlein et al., 2018). ASM overexpression in Jurkat cells substituted for CD28 engagement with regard to NF-κB activation, suggesting that ASM activation and extrafacial ceramide release might be favorable for T-cell activation (Boucher et al., 1995). This was supported by studies revealing attenuated CD8^+^ cell proliferation and activation in a graft-versus-host model in ASM-deficient mice and interference of pharmacological and genetic ASM activation with activation and functional differentiation of human naïve CD4^+^ T cells stimulated with α-CD3/CD28 coated beads *in vitro* (Rotolo et al., 2009; Bai et al., 2015). In contrast, costimulation of primary T cells on a planar surface was associated with retention of NSM2 activity, but not of ASM. This suggested the ASM activity might be promoted by ligation of CD28 alone to prevent T-cell activation and that activation of the enzyme might rather be silenced upon antigen recognition (Mueller et al., 2014; Bortlein et al., 2018). Interestingly, uncontrolled inflammatory T helper 1 (Th1) and Th17 responses were observed in ASM-deficient mice in a pathogen-driven colitis model (Meiners et al., 2019). Taking into account the differential effect of genetic ASM deficiency on T-cell subsets in this and another study (Hollmann et al., 2016) (detailed below), there is an obvious need to ultimately resolve the role of ASM in T-cell viability, activation, and expansion before this pathway can be explored for therapeutical intervention.

As referred to above, ASM activity and extrafacial ceramide release upon CD95 or TNFR ligation interfered with T-cell activation, and homeostatic ASM activity dampened their T*_*reg*_* function, further suggesting a negative rather than supportive role of this enzyme in T cells (Lepple-Wienhues et al., 1999; Church et al., 2005). Among other potential mechanisms, cross-regulation at the level of outer and inner leaflet membrane microdomains might contribute to ASM-mediated interference with TCR signaling. Hence, integrity of extrafacial nanodomains enriched in sphingomyelin was found to play a crucial role in triggering the phosphatidylinositol-3 kinase/Akt signaling pathway at the inner membrane leaflet by facilitating Akt recruitment and activation upon phosphatidylinositol-3,4,5-triphosphate accumulation (Lasserre et al., 2008). Remarkably, exogenously added sphingomyelinase prevented formation of inner leaflet nanodomains and, thereby, Akt activation, which is a central effector in costimulation. Moreover, ASM activation may result in collapse and paralysis of the T-cell actin cytoskeleton as revealed by MV surface contact (Gassert et al., 2009). In contrast to stimulated conventional T cells, the steady-state activity of ASM appears to negatively regulate the T*_*reg*_* compartment because this T-cell subset was represented at higher frequencies in ASM-deficient mice at the expense of conventional CD4^+^ T-cell (T*_*conv*_*) frequencies (Hollmann et al., 2016; Zhou et al., 2016).

## Impact of Sphingomyelinases on T-Cell Differentiation and Effector Function

The role of ASM in differentiation of the CD4^+^ T*_*conv*_* subset following clonal expansion is as yet unclear. While CD4^+^ T cells of ASM-deficient mice effectively differentiated into Th1 and Th17 cells with comparable kinetics and magnitude as their wild-type kins, ASM deficiency abrogated *in vitro* differentiation of human CD4^+^ T cells into Th17 cells (Tischner et al., 2011; Bai et al., 2015). Sphingomyelinase activity-dependent regulation of T*_*conv*_* effector functions was clearly identified at the level of cytokine release. Mechanistically, this may relate to the documented impact of both ASM and NSM2 on the vesicular secretory pathway alluded to above (Stoffel et al., 2016), with specificities for individual cytokines/effectors being noted. Revealing the importance for ASM in IL-2 release, this cytokine was produced to lower amounts by ASM-deficient splenocytes and CD4^+^ T cells (Stoffel et al., 1998; Tischner et al., 2011), and this should have an obvious effect on the activity of other T-cell subsets depending on this cytokine. In fact, ASM-deficient effector memory T cells reacquired resistance against glucocorticoid-induced cell death upon IL-2 addition, and reduced levels of IL-2 secretion by ASM-deficient CD4^+^ T cells contributed to impairment of primary CD8^+^ T-cell responses in mice infected with lymphocytic choriomeningitis virus (LCMV) (Herz et al., 2009; Tischner et al., 2011). In this model, viral clearance was less efficient in the absence of ASM with impairment of IFN-γ production and full degranulation of CD8^+^ T cells being strongly affected. While ASM might control IFN-γ release [as that of IL-17 in human T cells (Bai et al., 2015)] in this setting *via* its impact on the secretory pathway, defective degranulation was attributed to lack of membrane tension as required for efficient expulsion in the absence of ASM (Herz et al., 2009).

A role for NSM2 in the development and differentiation of the T-cell compartment has not yet been established. NSM2 catalyzes formation of exosomes which, as efficient subcellular vectors, would be expected to take part in regulating release of effector molecules from T cells (Trajkovic et al., 2008). Thus, NSM2-dependent unidirectional exosomal transfer of micro-RNAs from T cells to APCs was reported (Mittelbrunn et al., 2011), and this mechanism may also apply to transfer of coinhibitory micro-RNAs, which are released from T*_*reg*_*, thereby contributing to T*_*reg*_*-mediated suppression (Okoye et al., 2014). Again not yet directly proven, NSM2 may take part in T*_*reg*_* TCR activation upon engagement of auto-antigens and thereby contribute to survival and replenishment of the T_*reg*_ pool. As pointed out above, ASM activity has a significant impact on the T*_*reg*_* compartment that is increased upon both genetic and pharmacological inhibition of the enzyme (Hollmann et al., 2016; Zhou et al., 2016). Basal ASM activity and levels of accumulated ceramides measured in T*_*reg*_* cells exceed those of T*_*conv*_* cells, possibly reflecting the role of ASM in T*_*reg*_* survival, which is highly dependent on CD28 signaling (Hollmann et al., 2016). Moreover, Akt ser473 phosphorylation and Rictor levels were reduced in ASM-deficient T*_*reg*_*, indicating that the enzyme controls metabolic activity in these cells (Zhou et al., 2016). In addition to their frequency, ASM also negatively regulates T*_*reg*_* function because their suppressive activity and CTLA-4 turnover is enhanced in the absence of Asm. Importantly, enhancement of T*_*reg*_* activity upon ASM depletion was reflected by reduction of MV-specific CD8^+^ T cells in spleens, lymph nodes, and brains of experimentally infected animals and, thereby, enhancement of viral central nervous system (CNS) infection (Hollmann et al., 2016). ASM targets downregulating T_*reg*_ activity are undefined as is the role of ASM catalyzed ceramide release in this process. Curiously, ceramide levels were found even increased in ASM-deficient T cells, including T*_*reg*_* (Horinouchi et al., 1995; Hollmann et al., 2016; Schuchman and Desnick, 2017), indicating that compensatory activities act to modulate this pool. A recent study provided clear evidence that ceramide accumulation is particularly important in T*_*reg*_* metabolism, and function is driven by Foxp3 activity (Apostolidis et al., 2016; Kasper et al., 2016). The latter suppressed sphingomyelin synthase 1 (SMS1) expression and, thereby, conversion of ceramide into sphingomyelin. Accumulated ceramides promoted PP2A activation by trapping its inhibitory factor SET. Thereby, mTORC1 activity was downregulated while Foxp3 expression was stabilized in T*_*reg*_*, and their suppressive activity was enhanced. ASM-catalyzed ceramide release obviously had the opposite effect on T*_*reg*_* function (Hollmann et al., 2016; Zhou et al., 2016), and kinetics, magnitude, or compartmentalization of enzyme activity and/or ceramide release may contribute.

## Sphingomyelinases Take Part in but Are Not the Sole Players in Modulating T-Cell Biology at the Level of Sphingolipid Metabolism

As introduced earlier (Figure 1), biosynthesis and metabolization of sphingolipids is a highly dynamic process. Though this review has focused on the activity of sphingomyelinases and subsequent ceramide production, virtually all enzymes acting to define membrane sphingolipid composition are therefore important in cellular responses, also T cells and selected examples will be briefly considered here.

Thus, ceramide species generated by the activity of ER-resident ceramide synthases (CerS1–CerS6) differing in acyl chain length specificity (C_14_ to C_26_). These are expressed in a tissue-specific manner, with CerS2 and CerS4 being most abundant in lymphatic tissues and in leukocytes, thereby defining the accumulation of intermediate (C_18_–C_20_) or long or very long chain (C_20_–C_26_) ceramides as building blocks (Levy and Futerman, 2010; Stiban et al., 2010). Studies mainly conducted in CerS2 mice revealed the importance of especially very long chain sphingolipids (VLC-SLs) in immune cell functions. This may occur at the level of membrane microdomain composition as shown in liver cells, and neutrophil receptor sorting, signaling, or stability of receptors in lipid rafts was affected in the absence of CerS2 (Park et al., 2013; Barthelmes et al., 2015). In the T-cell compartment, CerS2 has been found to facilitate thymocyte egress by its ability to regulate S1P gradients, and as key to production of VLC-SLs for development and homeostasis of invariant NKT cells, for which they serve as activating ligands (Rieck et al., 2017; Saroha et al., 2017).

As already alluded to above, glycosphingolipids are major constituents of lipid rafts, the role of which in T-cell development, activation, and signal initiation has been amply documented (for a review, see Wu et al., 2016; Nakayama et al., 2018) and will not be reiterated here. Of note, T-cell subsets substantially differ with regard to their membrane composition of membrane gangliosides. This proved to be critical for their function and has been suggested to link to organization of specific membrane microdomains by the respective gangliosides (Inokuchi et al., 2013). The importance of another ceramide derivative, C1P, generated through the activity of the ceramide kinase, for T cells is less well investigated. In contrast to what has been reported for ceramide accumulation, increased levels of C1P were found to activate Ca^2+^ mobilization *via* store-operated channeling in Jurkat cells (Church et al., 2005; Colina et al., 2005).

Ceramide accumulation due to sphingomyelin breakdown is counter-regulated by the activity of two enzyme species, ceramidases and sphingomyelin synthases, giving rise to sphingosine or sphingomyelin, respectively (Figure 1). In line with its ability to metabolize ceramide, genetic depletion of acid ceramidase increased overall ceramide levels, while its overexpression promoted cell growth as analyzed in non-lymphoid cancer cells (Saad et al., 2007; Brodlie et al., 2010). More recently, exogenous application of acid ceramidase was found to cause Akt kinase activation in Jurkat cells, and, however, affected their expansion. The latter was suggested to relate to the inability of the added ceramidase to promote activation of sphingosine kinase and, thereby, production of S1P in this system (Baduva et al., 2019). The ability of this particular bioactive sphingolipid to substantially regulate survival, trafficking, and activity of immune cells including T cells is well established, and with FTY720, a drug targeting S1P activity is in clinical use. Though it is therefore of critical importance to fully appreciate the relevance of the sphingolipid metabolism on T cells, it is far beyond the scope of this review to extend on this topic (for excellent reviews, see Pyne and Pyne, 2010; Stepanovska and Huwiler, 2019).

SMS1 and 2 both localize to the Golgi compartment, while SMS2 is also found at the plasma membrane. As being crucial for *de novo* sphingomyelin synthesis, they regulate availability of this sphingolipid (and thereby glycosphingolipids) for organization and integrity of lipid rafts. Therefore, their activity is also of crucial importance in the regulation of T-cell biology, and this has been highlighted in studies revealing that TCR signaling, migration, and apoptosis are highly sensitive to the absence of SMS1 (Jin et al., 2008; Lafont et al., 2010; Asano et al., 2012). Because its catalytic site locates to the extrafacial leaflet of the plasma membrane, SMS2 can directly oppose ASM activity and, thereby, ceramide accumulation by regenerating sphingomyelin (Milhas et al., 2010a). The role of SMS2 in T-cell development and activation has, however, not yet been investigated. Curiously, SMS2 rather than SMS1 was found to be involved in HIV-1 env-mediated membrane fusion with T cells, and this activity was attributed to the SMS2 protein itself rather than to its enzymatic activity (Hayashi et al., 2014).

## Outlook

Common to that of other cell types and compartments, the spatiotemporal resolution of the sphingolipid metabolism will crucially advance our understanding of the impact of this system on T-cell activation, trafficking, differentiation and effector functions, and, thereby, in protection or pathophysiology. At a cellular level, this, for instance, applies to the enzyme NSM2, which, in non-T cells, appears to shuttle between the plasma membrane, endo-lysosomal, Golgi, and nuclear membranes (Albi et al., 2008; Cascianelli et al., 2008; Clarke et al., 2008; Trajkovic et al., 2008; Milhas et al., 2010b; Airola and Hannun, 2013), where conceivably the sphingomyelin breakdown may differ in kinetics, efficiency, and physiological responses. The advent of bio-orthogonally mono-, bi-, or tri-functionalized sphingolipids in conjunction with targeted enzymes has enabled us and others to investigate trafficking and compartment-specific metabolization of sphingolipids (Haberkant et al., 2013, 2016; Hoglinger et al., 2014, 2017; Collenburg et al., 2016; Walter et al., 2016, 2017; Feng et al., 2018; Laguerre and Schultz, 2018), and if further advanced, this toolbox will allow to study the impact of compartment-specific enzyme activity and lipid localization on signaling and the metabolic fate of T cells. This also applies to detailed studies on membrane topology of sphingolipid metabolites being generated and/or accumulating at cytosolic or anticytosolic membrane leaflets and organizing membrane microdomains there, which will be a challenging task. Reagents and microscopical techniques allowing to resolve lipid association with membrane leaflets ideally also allowing for codetection and copurification of proteins continue to be developed (Heilemann et al., 2009; Collenburg et al., 2016; Burgert et al., 2017). In combination with mass spectrometry performed on protein complexes crosslinking to functionalized sphingolipids after photoactivation (Hoglinger et al., 2014, 2017; Haberkant et al., 2016), detailed analyses on the organization of functionally active membrane microdomains such as, for instance, lamellopodia or immune synapses, will become possible.

Mass spectroscopy-based analytical approaches have substantially increased the sensitivity to detect and quantify sphingolipids and, when coupled to imaging, enabled spatial resolution of sphingolipid classes accumulating in tissue specimens, for the time being, at the expense of sensitivity (reviewed in Luberto et al., 2019). If further advanced, this technology will be very instrumental in relating sphingolipid patterning to the architecture of lymphoid tissue and, ideally, cellular compartments therein. At an organismic level, inbred mouse strain deficient for or overexpressing sphingolipid metabolizing enzymes have provided important insight into the importance of this system also in T-cell biology (see above). Ubiquitous disruption of enzyme activity was often associated with the development of lipid storage or other severe diseases in mice, thereby precluding long-term analyses or—except for adoptive transfer approaches—hampered assignment of immunological alterations to a specific compartment *in vivo*. In conjunction with the progress made in evaluating the sphingolipid metabolism in T cells at a subcellular, cellular and tissue level, the recent advent of novel mouse strains allowing for cell-specific inducible expression or ablation of sphingolipid-modifying enzymes will doubtlessly enable the understanding of this system for T-cell biology and delineate targets and strategies for specific intervention.

## Author Contributions

All authors listed have made a substantial, direct and intellectual contribution to the work, and approved it for publication.

## Conflict of Interest Statement

The authors declare that the research was conducted in the absence of any commercial or financial relationships that could be construed as a potential conflict of interest.

## Footnotes

1 ASM and NSM2 in humans, Asm and Nsm2 in mice. As some studies cited investigated both human and mouse cells, we will use the nomenclature ‘ASM’ and ‘NSM2’ to facilitate reading.
